# Quantifying widespread hydrothermal chimneys on the East Pacific Rise flanks between 9°43′ and 57′N

**DOI:** 10.1126/sciadv.adv0788

**Published:** 2025-11-07

**Authors:** Jyun-Nai Wu, Ross Parnell-Turner, Daniel J. Fornari, Thibaut Barreyre, Jill M. McDermott

**Affiliations:** ^1^ Institute of Geophysics and Planetary Physics, Scripps Institution of Oceanography, University of California, San Diego, La Jolla, CA 92037, USA.; ^2^ Department of Geology and Geophysics, Woods Hole Oceanographic Institution, Woods Hole, MA 02543, USA.; ^3^ Geo-Ocean, CNRS, Univ Brest, Ifremer, UMR6538, F-29280 Plouzane, France.; ^4^ Department of Earth and Environmental Science, Lehigh University, Bethlehem, PA 18015, USA.; ^5^ Lehigh Oceans Research Center, Bethlehem, PA 18015, USA.

## Abstract

Hydrothermal circulation at mid-ocean ridges drives the exchange of heat and matter from Earth’s interior to the global ocean and supports deep-sea life. Away from the ridge axis, however, the spatial extent of hydrothermal discharge remains enigmatic. Using near-bottom data for a 25-kilometer-long section of the East Pacific Rise between 9°43′N and 9°57′N, we show that considerable hydrothermal flow occurs at variable distances from the ridge axis. Mapping the seafloor and water column along this segment using an autonomous underwater vehicle, we identified 448 candidate hydrothermal chimneys. More than half of them lie outside the axial summit trough, indicating that hydrothermal fluids discharge over a larger area than previously thought. Water column measurements show that >27% of mapped constructs are likely to be venting actively. Our results indicate that widespread active hydrothermal flow occurs over the near-axis region, with important implications for constraining total heat flux along mid-ocean ridges and for identifying previously unexplored benthic habitats.

## Teaser

Near-bottom mapping finds widespread active hydrothermal venting kilometers away from the axis along the East Pacific Rise 9°50'N.

## INTRODUCTION

Circulation of high- and low-temperature fluids through oceanic crust at mid-ocean ridges (MORs) plays a crucial role in heat and chemical exchange between the solid Earth and oceans (*1*). This first-order process accounts for 10% of all oceanic lithospheric heat loss (*2*–*5*) and leads to high-temperature “black smoker”–type vents and spires that support dynamic, ephemeral chemosynthetic habitats (*6*–*9*). While models for hydrothermal circulation address flow, partitioning, and recharge, the extent of diffuse, near-axis fluid circulation remains largely unconstrained because of sparse observational data (*6*, *10*–*14*). Seawater enters the seafloor either on- or near-axis where it gains heat from subsurface magma bodies below the ridge crest and subsequently flows vertically and laterally within the deep and shallow crust before being discharged as hydrothermal vent fluids (*6*, *15*, *16*). Hydrothermal fluids exit the seafloor in a continuum between high-temperature (>250°C) and diffuse, low-temperature fluids (~5° to 20°C) (*17*–*19*). These two types of discharge play different roles in crust-ocean heat and chemical exchanges, resulting in variable vent fluid compositions and alteration-based mineral assemblages in the host volcanic rocks (*17*, *20*).

The spatial extent of hydrothermal venting (area, *A*) plays a critical role in determining total heat flux, as it directly scales heat flux estimates (Eq. 1 in Materials and Methods), and influences the balance between diffuse and focused flow in hydrothermal models (*20*–*22*). This key parameter remains virtually unconstrained because of a lack of sufficiently high-resolution bathymetric data required to identify potential vent chimneys and related hydrothermal features. As a result, estimates of hydrothermal flux are limited to highly localized measurements around individual vent sites, which are not representative of spatial distribution and quantification of hydrothermal discharge at the ridge-segment scale (*17*, *20*, *23*–*27*). Progress has been made in constraining hydrothermal flux around individual hydrothermal chimneys or vent fields covering several square kilometers, but multifield, segment scale estimates remain elusive (*21*, *22*, *28*). In addition, although hydrothermal plume measurements show evidence for pervasive diffuse flow (*29*), measurements constraining the spatial extent of diffuse flow are limited to within a few hundreds of meters from the ridge axis (*22*, *24*). While it is possible to calculate large-scale hydrothermal heat output from water-column geochemistry and temperature anomalies of hydrothermal plumes, such estimates are highly sensitive to density gradients in ambient seawater and nonbuoyant plume height, leading to uncertainties of at least a factor of 2 (*23*, *30*, *31*).

**Fig. 1.**
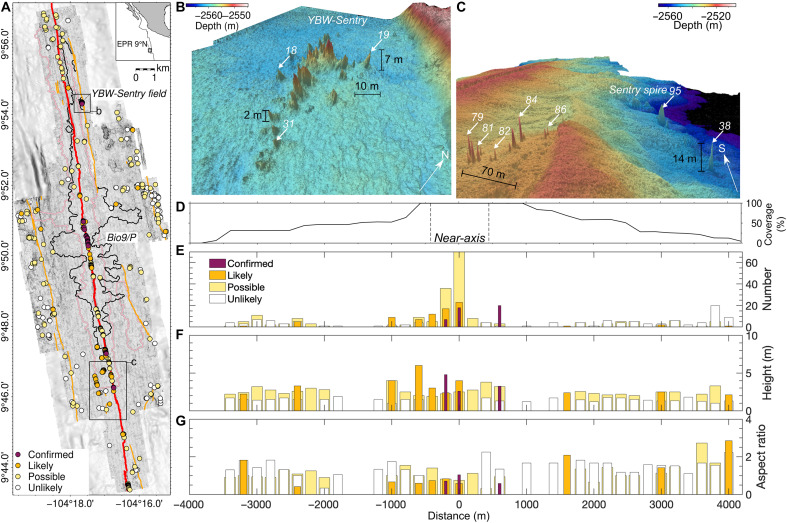
Bathymetric constructs mapped between 9°43′ and 57′N, EPR. (**A**) One-meter resolution bathymetric data collected by AUV *Sentry*; black outline is the extent of 2005 to 2006 eruption (*46*); pink outline is axial magma lens (*39*, *44*, *76*, *77*); orange lines are normal faults (*55*); red line is AST; filled circles are 448 bathymetric constructs identified, colored by likelihood of active modern venting. (**B** and **C**) Three-dimensional views of chimneys at YBW-Sentry and Sentry Spire vent fields, respectively; white arrows in lower right indicate view directions; numbered white arrows indicate construct index number (figs. S1 to 15). Vertical exaggeration is 3 and 7 for (B) and (C), respectively. (**D**) Integrated bathymetric data coverage (%) across-axis; dashed lines indicate near-axis region, defined as being <400 m from AST; (**E** to **G**) number, median height, and aspect ratio of mapped constructs versus distance from AST, respectively.

The 9°50′N segment of the East Pacific Rise (EPR) is well-studied and volcanically active. It hosts hydrothermal vent fields both on- and near-axis, with documented eruptions occurring every ~15 to 20 years (Fig. 1, A and B) (*14*, *32*–*35*). Two recent eruptions (in 1991 to 1992 and 2005 to 2006) before the most recent, 29 April 2025 eruption (*36*), were investigated during numerous field experiments that included hydrothermal fluid sampling, while seismicity and fluid temperatures were monitored continuously during the 2005 to 2006 eruption cycle (*34*, *35*, *37*, *38*). Volcanic activity at EPR 9°50′N is driven by a seismically imaged 0.5- to 1.7-km-wide axial magma lens, located 1500 to 1800 m below the seafloor, extending nearly continuously along the entire segment (*39*–*44*). While most of the observations and experiments at 9°50′N have been done within the axial summit trough (AST), recent near-bottom surveying led to the discovery of the YBW-Sentry vent field (*45*). Located ~750 m from the AST, observational data indicate that hydrothermal chimneys in the YBW-Sentry field may have been influenced by lava flows associated with the 2005 to 2006 eruptions. Lava flows erupted in 2005 to 2006 from axial fissures further south along the EPR crest near 9°51′ to 53′N were channeled along the north flank of the ridge crest and contained by the ~5- to 7-m-high first inward-facing fault scarp on the east side of the axis that extends between ~9°51′N and 9°57′N (*35*, *46*). Some of these flows may have onlapped the base of active and inactive chimneys at YBW-Sentry.

To evaluate the segment-scale hydrothermal budget and distribution of vent spires, we mapped chimney-like constructs across a segment of the EPR between 9°43′N and 9°57′N using the autonomous underwater vehicle (AUV) *Sentry*. We use coincident water column observations made by the AUV to evaluate the probability that constructs are actively venting. Using these observations, combined with seafloor imagery from the remotely operated vehicle (ROV) *Jason* and human-occupied vehicle (HOV) *Alvin*, we quantify the presence of hydrothermal discharge sites over a large area along and across the EPR axis and provide estimates of likely activity as well as distinguishing whether the features are hydrothermal or volcanic in origin.

## RESULTS

### Spire mapping

**Fig. 2.**
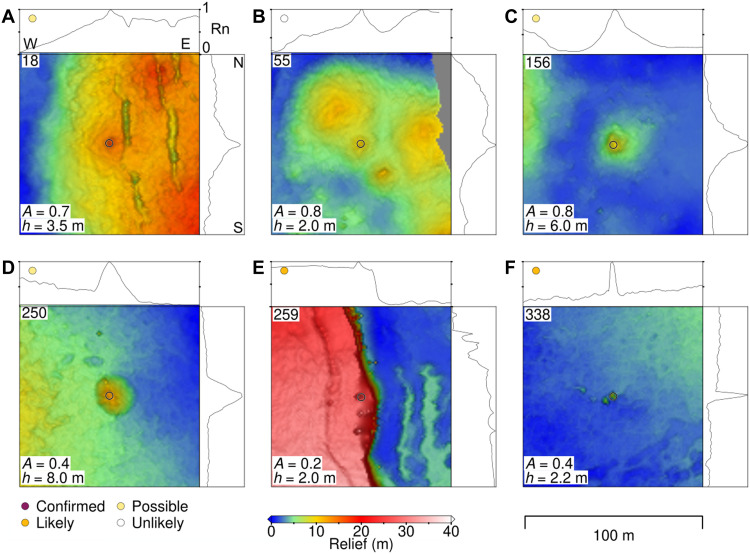
AUV *Sentry* bathymetric maps of seafloor constructs. (**A**) A 100 m–by–100 m map of a construct adjacent to volcanic fissures, top/right panels show normalized bathymetric profiles (Rn, normalized relief) across the construct from west to east and north to south across the center, respectively. Colored dot and number at top left corner of top panel show the category and index number, respectively; open circles on map indicate construct; aspect ratio (A) and local relief (*h*) noted in lower left. (**B**) Construct located on top of a cone. (**C** and **D**) Isolated constructs. (**E**) Constructs located near faults. (**F**) Constructs located on the edge of lava lobes or flow fronts. A full list of constructs and their locations is provided in figs. S1 to S15 and spreadsheets in the Supplementary Materials.

Chimney-like constructs were mapped using 1-m–resolution bathymetric data collected during 20 AUV dives along a 25-km-long by 8-km-wide segment of the EPR (*46*). We identified 448 constructs with >2 m vertical relief and aspect ratio >0.1 (i.e., ratio between construct relief and basal radius; Fig. 1 and figs. S1 to 15). These geometrical screening parameters removed smaller constructs likely to be of volcanic origin, such as pillow mounds and hornitos. Of the 448 spire-like constructs meeting these geometrical criteria, 332 (72%) are located outside the 2005 to 2006 eruption area (Fig. 1A), and 201 (~45%) are >400 m from ridge axis, which is the median distance that 2005 to 2006 eruptions extended east and west from the AST. Many of these potential vent constructs are proximal to normal faults on either flank of the ridge axis and away from ridge axis, despite the bathymetric data coverage being increasingly discontinuous at distances >1000 m from the axis (Fig. 1, D and E). Only a few constructs are located within the 2005 to 2006 eruptions’ area yet outside the AST (Fig. 1A), suggesting that newly erupted flows pave over small preexisting vent constructs and that those observed today were likely formed in situ, outside the confines of the AST. Potential vent constructs are found in a variety of settings, including near volcanic fissures (Fig. 2A), cones (Fig. 2B), sheet flows (Fig. 2, C and D), fault scarps (Fig. 2E), and along the edge of lava lobes (Fig. 2F).

Constructs located within ~250 m of the youngest near-axis normal fault are often associated with only one anomaly along, potentially due to either other anomalies being below detection limits or vent inactivity (Fig. 1A). Alternatively, hydrothermal discharge near these faults could undergo more turbulent mixing by bottom currents, enhanced by increased local seafloor roughness resulting in weak, heterogenous plumes (*47*–*51*).

### Likelihood of active venting and vent field area estimates

**Fig. 3.**
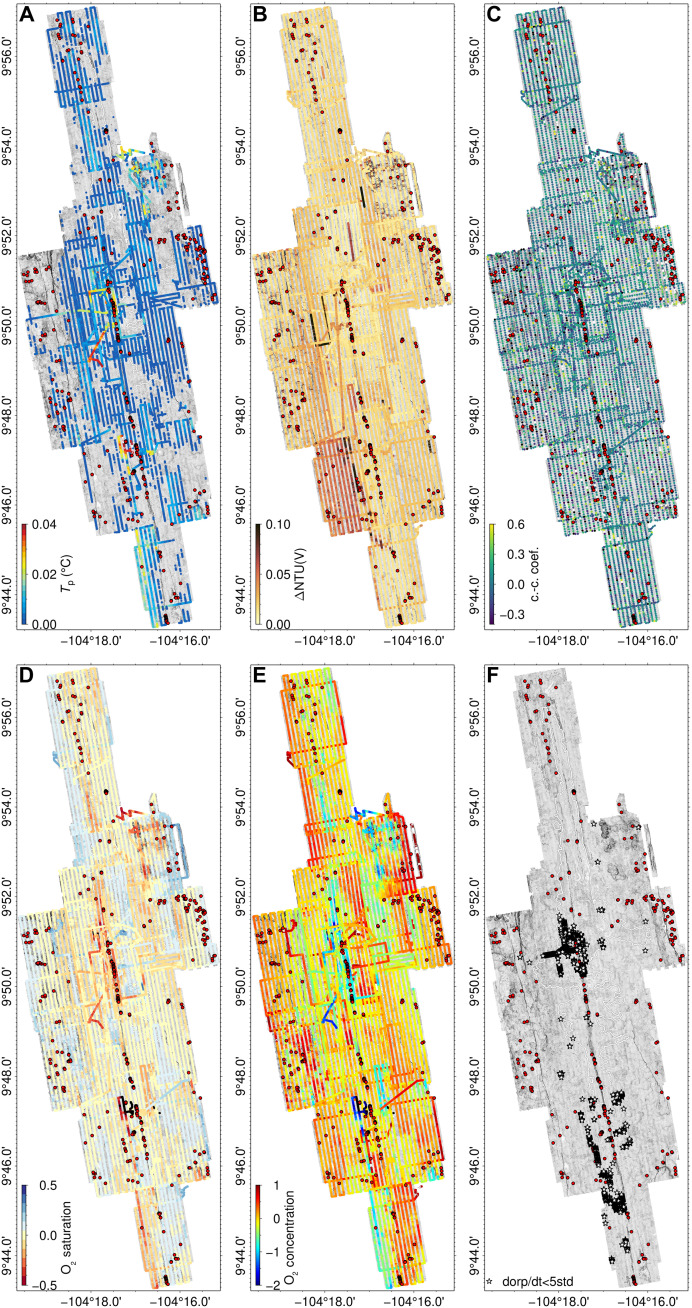
Water column measurements from AUV *Sentry*. (**A**) Potential temperature (*T*_p_, °C), (**B**) turbidity (∆NTU, V), (**C**) correlation coefficient between *T*_p_ and V, (**D**) oxygen saturation, (**E**) oxygen concentration, and (**F**) anomalous time derivative of ORP (dORP/dt) collected in near-bottom water (<80 m from seafloor).

**Fig. 4.**
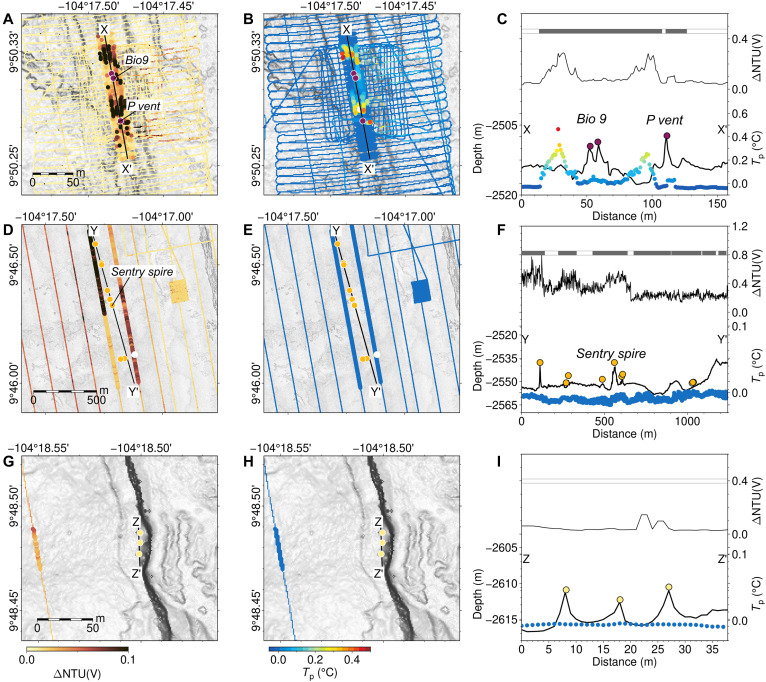
Water column data used as indicators of active venting. (**A** and **B**) Turbidity and potential temperature measured by AUV *Sentry* at altitude of 20 m, respectively, above confirmed active Bio9 and P vents (R/V *Atlantis* expedition AT42-06, 2018); (**C**) bathymetric profile X to X′ across Bio9 and P vents (thick black line) with coincident potential temperature, turbidity, and ORP anomalies (thin black line, shaded dots, and gray bars, respectively). (**D** to **F**) As above but measured at altitude of 65 m, for the likely active Sentry Spire vent located ~700 m west of the AST near 9°46.3′N; (**G** to **I**) as above but measured at altitude of 65 m, for possible active vents located 4 km west of the spreading axis.

**Fig. 5.**
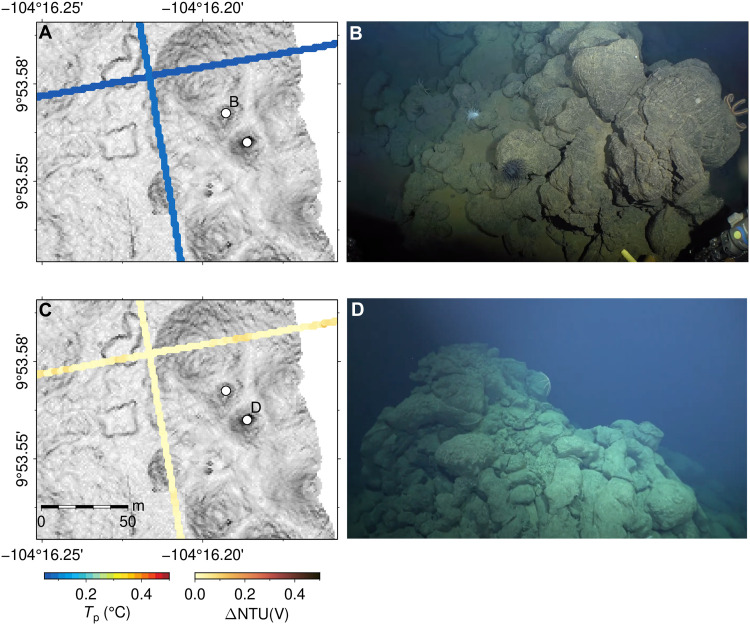
Examples of constructs confirmed to not be chimneys by submersible vehicle imagery near 9°53′N. (**A** and **C**) Potential temperature and turbidity measured by AUV *Sentry* at altitude of 80 m, respectively, showing two “unlikely active” constructs located in NE volcanic area from 9°50′N ridge axis. (**B** and **D**) Still images, looking NW, acquired by ROV *SuBastian* dive 559 (R/V *Falkor* expedition FKt230629, July 2023) showing the volcanic mounds and lava pillars, confirming that the constructs are not hydrothermal chimneys.

To quantitatively evaluate the likelihood of active hydrothermal venting at each of the 448 mapped constructs, we used coincident, near-bottom water column properties measured by *Sentry* (Fig. 3). As hot, focused vent fluids mix with ambient seawater, minerals precipitate to form warm, particle-rich plumes that contain abundant reduced chemical compounds (*18*, *52*, *53*), leading to measurable anomalies in potential temperature, turbidity, and oxidation-reduction potential (ORP). Considering the aggregated water column anomalies (see the Supplementary Materials and Materials and Methods) for each chimney-like construct, we assigned them to one of four groups reflecting their potential for active venting: (i) active vents, confirmed by in situ sampling or measurement of high-temperature fluids via submersible (Fig. 4, A to C); (ii) likely active constructs with anomalies in two or more water column parameters (Fig. 4, D to F); (iii) possibly active constructs with anomalies in only one water column parameter (Fig. 4, G to I); and, (iv) unlikely active constructs with no detectable water column anomaly (Fig. 5). Of 448 chimney-like constructs mapped, 123 were categorized as confirmed or likely active (i.e., groups 1 and 2), while 209 were determined to be possibly active (group 3). The remaining 116 features fall into category 4 and are unlikely to be active or may be of volcanic constructional origin.

**Fig. 6.**
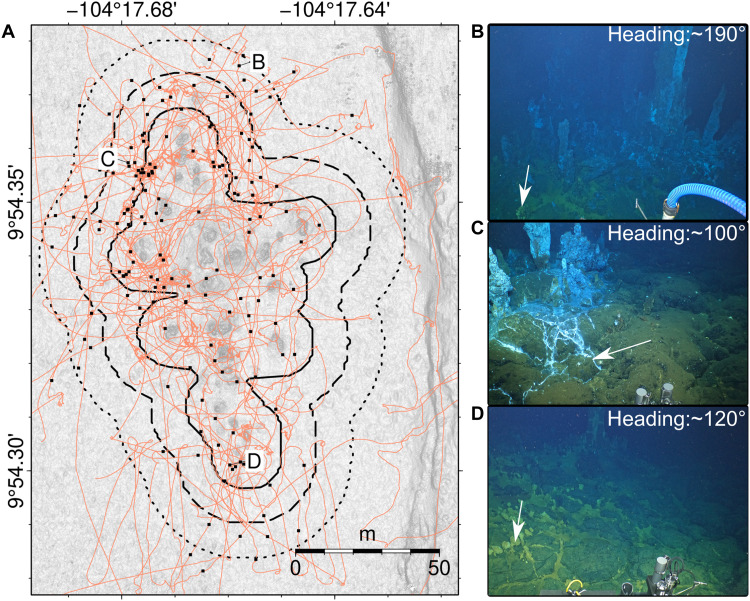
Evidence for diffuse venting around YBW-Sentry vent field. (**A**) A 0.25-m resolution bathymetric map; solid/dashed/dotted lines are minimum/most likely/maximum areas of diffuse venting, respectively; black dots indicate locations with evidence of diffuse flow; orange lines are vehicle tracklines with seafloor imagery. (**B** to **D**) Still photographic images collected by ROV *Jason* and HOV *Alvin*; evidence of diffuse flow indicated by white arrows.

On the basis of our combined mapping and water column data analysis approach (see the Supplementary Materials and Materials and Methods), we find that the focused flow area, *A*_f_, for on-axis vent clusters in the segment between 9°43′N and 9°57′N is 0.3 ± 0.03 m^2^, and is 0.24 ± 0.02 m^2^ for the YBW-Sentry vent field, which is ~750 m east of the current axis at ~9°54′N. By comparison, Ramondenc *et al.* (*24*) estimated *A*_f_ to be 0.09 m^2^, which is considerably lower than our calculated estimate, due to their approach, which omitted a large area of seafloor where many active vent orifices are known to exist. Assuming that diffuse flow is only associated with confirmed-active chimneys (i.e., group 1; Material and Methods), the diffuse flow area, *A*_d_, associated with on-axis vents is 52.7 ± 26.2 m^2^ and 52.0 ± 28.4 m^2^ for the YBW-Sentry field (Fig. 6). These estimates are minima since diffuse flow also occurs in other places in addition to active vents [e.g., Lucky Strike vent field on the Mid-Atlantic Ridge near 37°N (*21*)].

## DISCUSSION

### Distribution of chimney-like constructs and near-axis hydrothermal activity

We find that numerous chimney-like constructs are found in the near-axis region of the EPR crest between 9°43′N and 9°57′N, at least 37% of which (*n* = 332) are likely to be actively venting. Constructs are sparsely distributed within the area covered by lava flows erupted in 2005 to 2006 and outside the AST, implying that they are vulnerable to being paved over during eruptions. The presence of near-axis constructs in the study area indicates that considerable hydrothermal discharge must occur up to several kilometers from the ridge axis and beyond the confines of the AST. After downwelling into the crust, sea water obtains heat from subaxial or near-axis magma lenses and then migrates along damage zones created by near-axis faults to reach the seafloor. The presence of inflated lava plateaus and volcanic fissures overlying near-axis magma lenses also agrees with the concept of near-axis heat exchange and fluid flow (*33*, *54*, *55*). Our results suggest that near-axis crust remains hot enough to support hydrothermal flow, consistent with evidence for relatively slow cooling of oceanic crust at fast-spreading ridges (*56*).

**Fig. 7.**
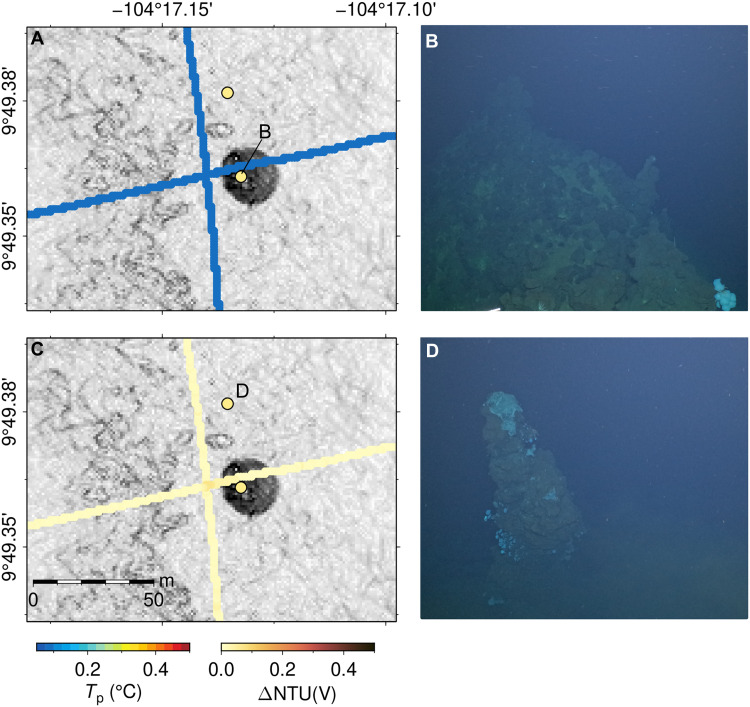
Examples of constructs confirmed to be inactive chimneys by submersible vehicle imagery near 9°49′N. (**A** and **C**) Potential temperature and turbidity measured by AUV *Sentry* at altitude of 80 m, respectively, showing two possibly active constructs located east of AST. (**B** and **D**) Still images, looking SE, acquired by HOV *Alvin* during dive AL5243 (R/V *Atlantis* expedition AT50-21, March 2024) showing extinct chimneys, confirming that the constructs are hydrothermal chimneys but not actively venting at time of mapping.

Our statistical approach for evaluating the likelihood of venting activity can be tested with reconnaissance observations. For instance, an ~8-m-tall construct located east of AST, considered to be “possibly active” in our analysis (Fig. 2D), was subsequently determined to be an extinct sulfide chimney when visited with *Alvin* in 2024 during cruise AT50-21 (Fig. 7). Although no examples of “likely active” constructs have yet been directly confirmed to have active venting, most “confirmed” chimneys exhibit water column anomalies. About 75% of the chimneys, other than the ones in the YBW-Sentry field, exhibit more than two water-column anomalies, while the remaining ones show only one anomaly. Notably, only a single water column anomaly was detected in the vicinity of the active YBW-Sentry field, possibly because of perturbations of its plume by bottom currents and topographic effects caused by the nearby fault scarp [e.g., (*47*–*51*)]. Our combined statistical and quantitative analysis of observational and near-bottom water properties data suggest that ~50 likely active constructs may be actively venting along this fast-spreading EPR segment and may represent previously unexplored chemosynthetic habitats. This result further highlights that the extent of hydrothermal activity along MOR segments is underestimated given the generally axially focused field studies carried out to date (*32*).

### Hydrothermal heat flux estimates along the EPR 9°43′ to 57′N segment

By combining the scaling factor of diffuse flow area (despite its large uncertainty), with the calculated vent field area, we can estimate the minimum hydrothermal heat flux *H*, for focused and diffuse flow (*H_f_* and *H_d_*), given by

**Fig. 8.**
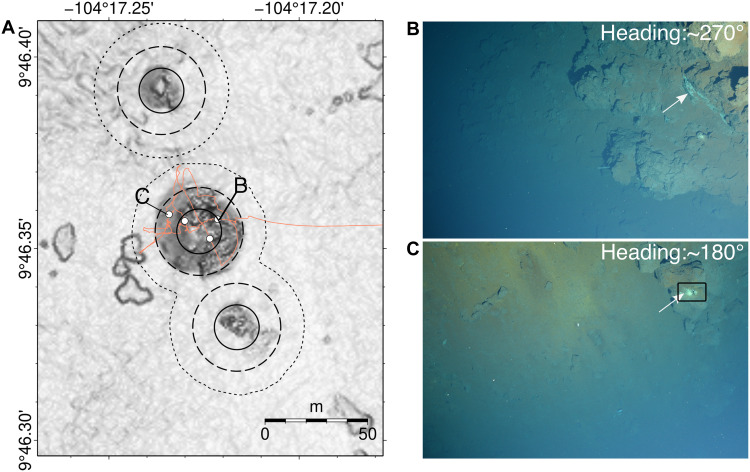
Evidence for diffuse venting around Sentry Spire vent field. (**A**) 1.0-m resolution bathymetric map of Sentry Spire vent field; solid/dashed/dotted lines are minimum/most likely/maximum areas of diffuse venting, respectively; white dots indicate locations with evidence of diffuse flow; orange lines are vehicle tracklines with seafloor imagery. (**B** and **C**) Still photographic images collected by ROV *Jason*; evidence of diffuse flow indicated by white arrows; black box shows portion of image frame occupied by evidence for diffuse flow, *B* = 0.005.

H=cpρfvTA (1)

where $c_{p}$ , $\rho_{f}$ , v , T are the specific heat, fluid density, fluid velocity, and exit-fluid temperature, respectively, and A is the cross-sectional area of flow, defined separately for focused and diffuse flow. Focused vent fluid temperature varies considerably during the eruption cycle at the 9°50′N segment, with in situ measurements made between 1991 and 2008 ranging between 340° and 9°C (*45*, *57*). Exit-fluid velocity measured in 2004 and 2011 ranges from 0.25 ± 0.1 m/s at vent chimneys in the AST near 9°50′ to 51′N (*24*, *30*). We find that focused flow *H*_f_ ranges from 140 ± 66 MW, the upper range being much larger than previous estimates of 42 and 16 MW obtained from direct measurements and from modeling, respectively (*20*, *24*). For diffuse flow, *H*_d_, assuming diffuse fluid temperatures between 13° and 5°C, and diffuse flow velocities ranging between 0.06 and 0.01 cm/s from earlier estimates (*24*, *30*), we find that *H*_d_ varies from 349 ± 226 MW. This estimate is a lower bound and is consistent with a conservative previous estimate of 285 MW along this segment (*24*). On the basis of observations of diffuse flow around chimneys that are not actively venting but having likely active water column anomalies (Fig. 8), we also applied the diffuse flow area calculation to the likely active constructs group. This scenario yields *H*_d_ = 2626 ± 1707 MW which is, perhaps, a more realistic estimate considering that the partitioning between focused and diffuse flow [*H_d_*/(*H_f_ + H_d_)* = 82 to 98%] is more comparable to values found at other hydrothermal systems on the slow-spreading Mid-Atlantic Ridge and intermediate-spreading Juan de Fuca Ridge [e.g., Lucky Strike; ~95% and ASHES; 67 to 93%, respectively; (*20*, *26*, *27*, *58*, *59*)].

The difference between observed conductive heat flux and heat flux derived from a cooling model has been used to indicate the hydrothermal heat flux (*4*, *23*). A crustal cooling model of a 4-km-wide ridge postulates that the upper crust partially forms by crystallization in a gabbro glacier and the lower crust by partial crystallization in situ (*60*), providing a heat flux estimate of ~50 MW/km at 110 mm/year spreading rate (*61*, *62*). For this 25-km-long segment, the heat flux from cooling is ~1250 MW, which is roughly consistent to upper bound of our total conservative heat flux estimate (489 ± 235 MW), assuming that most heat extraction is from hydrothermal discharge. This crustal cooling model considers only a 4-km-wide MOR crest, whereas our estimate represents an ~8-km-wide crest. By applying the half-space cooling model (*4*), the thermal cooling model of a 25 km–by–8 km ridge segment yields a heat flux estimate of ~2300 MW, which is four times larger than the upper bound of our conservative estimate. In a more comparable scenario with other segments (e.g., Lucky Strike at the Mid-Atlantic Ridge and ASHES at Axial Seamount on the Juan de Fuca Ridge), our estimate yields a total heat flux of 2766 ± 1708 MW, which agrees better with the cooling model and hybrid hydrothermal circulation model of 2250 MW for 25-km segment (*6*) regardless of uncertainty. Our estimate of widespread hydrothermal spires extending 4 km over an area east and west of the AST is consistent with the presence of off-axis magma lenses [e.g., (*44*)] and raises the potential for previously undiscovered vent-supported ecosystems outside of the axis. Future field studies and models for hydrothermal discharge and chemosynthetic ecosystem development at other MORs should include broader, high-resolution AUV-based bathymetric mapping of off-axis terrain to capture features that may contribute to the substantial diffuse flow component of near-axis hydrothermal flux.

## MATERIALS AND METHODS

### Data acquisition

Near-bottom, multibeam bathymetric data were collected with AUV *Sentry* during 19 dives at altitudes of 65 or 80 m (trackline spacing 170 m; table S1) and one dive at 20 m above the seafloor during cruises in 2018, 2019, and 2021 (expeditions AT42-06, AT42-21, and RR2102, respectively). Bathymetric data were collected with a Reson 7125 echosounder in 2018, and a Kongsberg EM2040 echosounder in 2019 and 2021, both operating at 400 kHz (*46*). AUV navigation was calculated using a 300-kHz Teledyne Doppler velocity log (DVL) and a Sonardyne AvTrak2 ultrashort baseline acoustic positioning system, combined with an iXblue Phins inertial navigation system, and a Paroscientific 8B7000-I Digiquartz depth sensor. Data were processed using the open source MB-System software (*63*). During AUV-based multibeam surveys, water column measurements were taken coincidently. Temperature data were collected with a SeaBird 49 FastCAT conductivity-temperature-depth sensor with additional redundant sensors for temperature and conductivity, together with sensors for optical backscatter (Seapoint Turbidity Meter), dissolved oxygen (Aanderaa Optode, 4330F), oxidation reduction potential (PMEL-NOAA), pressure (Paroscientific, 8B7000-I), and an altimeter (Nortek, DVL500). Accuracy of the temperature sensor is 0.001°C, while the accuracy of optical backscatter and ORP measurements are 5 mV and 2 μ M, respectively. Other sensor specifications can be found in table S2.

Chimney-like seafloor constructs with approximately circular planform were initially identified in the bathymetric data by an analyst. A similar criterion has been used as part of a machine learning approach to detecting chimney-like structures in this study area (*64*). We estimated radius and relief of each construct, where radius is twice the distance between the local maximum depth of a construct base and the point of shallowest depth (i.e., peak). On the basis of previous observations at the EPR 9°50′N [e.g., (*32*)] and elsewhere [i.e., Juan de Fuca Ridge, Endeavor segment (*65*)], we expect hydrothermal chimneys to have relief >2 m and aspect ratio >0.1 (where aspect ratio is the ratio of relief:diameter). The relief criterion is determined by visual confirmation of active venting from vents that are >2 m in relief around the YBW-Sentry field (Fig. 1B). We identified 448 chimney-like constructs that meet these criteria for the analysis reported herein, 332 of which are considered likely or actively venting. This result identified more constructs than previous picks using the machine learning approach (*64*), which used a stricter threshold for picking.

### Water column anomalies

**Fig. 9.**
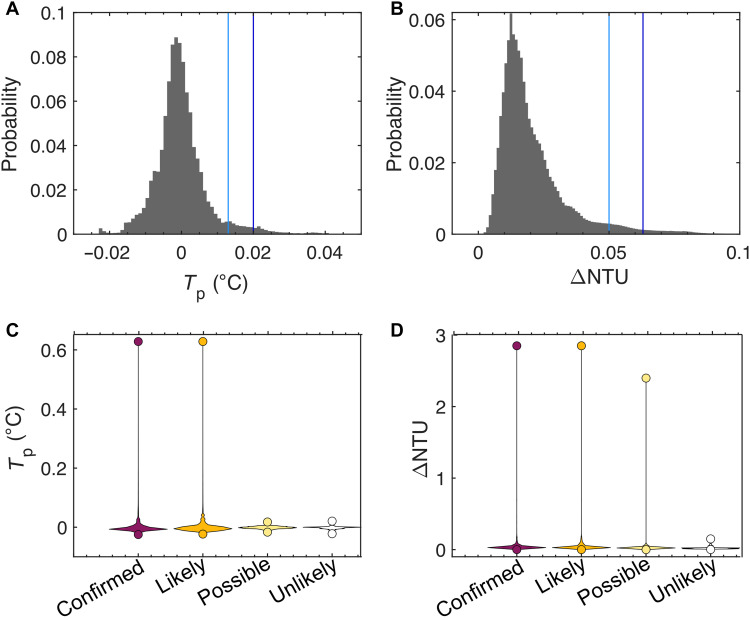
Water column statistics from AUV *Sentry*. (**A** and **B**) Histograms of all potential temperature *T*_p_ and turbidity, ΔNTU, measurements made during 20 *Sentry* dives across study area; light/dark blue lines are 95th and 98th percentiles, respectively; 98th percentile is the threshold used in this study to identify an anomalous measurement. (**C** and **D**) *T*_p_ and ΔNTU for a subset of measurements made within 170 m of constructs, grouped by likelihood of active venting; dots are minima/maxima extrema; colored histograms show distribution of all other values.

Potential temperature, ORP, and turbidity measured relative to background levels are commonly used to detect and map anomalous hydrothermal plumes in the water column (*66*–*71*). To define anomalous water column signals, we consider the statistical distribution of all measurements of temperature and turbidity in the study area. Following a conservative approach, we define the thresholds of detection for a hydrothermal plume to be measurements within the 98th percentile of the population (0.02°C and 0.063 NTU, Nephelometric Turbidity Units, respectively; Fig. 9). ORP anomalies were defined using the time derivative (dORP/dt), with anomalies defined as exceeding five times the standard deviation of the local 2-hour moving window. Oxygen saturation and concentration (Fig. 3, D and E) are not used as criteria for anomaly detection here since they can reflect physical processes, such as variations in salinity and temperature due to current activity and water mixing that do not relate to the presence of hydrothermal plumes [e.g., (*72*)]. For each individual construct, anomalies were analyzed across a radius equal to the survey track line spacing (170 m). Thus, for any construct, we evaluate water column anomalies measured along at least two adjacent survey lines.

### Vent field area determination

Although vent field area is a crucial scaling parameter in estimating hydrothermal heat flux, previously it has been either loosely defined at the segment-scale, or more accurately defined but only at the individual chimney scale [e.g., (*20*, *24*)]. To reduce the uncertainty in *A*, here, we determine the vent field area separately for focused flow, $A_{f}$ , and for diffuse flow, $A_{d}$ . Following previous approaches, we estimate $A_{f}\;$ using the geometry of orifices associated with active black smoker vents [e.g., (*24*)]. A total of 90 orifices across the study area were identified for measurement using still and video imagery acquired using the submersibles *Alvin* and *Jason*. Since each chimney structure is only partially imaged and can host multiple orifices, it is not possible to simply count and measure the number of imaged orifices per vent site. To minimize this potential underestimate bias, we used a bootstrapping approach to estimate that bias for a subset of 14 confirmed-active chimneys for which high-resolution imagery is available (i.e., those in group 1), and there is an average of two orifices per chimney structure (group 1). The focused flow area $A_{f}$ is given by

Af=nπr2 (2)

where *n* is the number of confirmed-active orifices (90), *r* is the mean circular vent orifice radius (4.4 cm), estimated by bootstrapping 1000 times from measurements of 37 individual orifices across the study area (table S3). Orifice radius size was estimated by visual comparison between orifices and instruments of known size in the same field of view on video and still imagery, consistent with approaches previously adopted (*24*).

For diffuse flow, we estimate the area of diffuse flow discharge, $A_{d}$ , using a combination of bathymetric mapping and imagery. Hydrothermal diffuse flow often occurs in association with clay minerals, weathered sulfide, and microbial mats, which leave a light yellow or white-mottled surface, and can concentrate fine-grained material into surface cracks in exposed lava crusts (*22*, *73*–*75*). These deposits were used as indicators of diffuse flow in still and video imagery collected during six submersible dives of ROV *Jason*, *Alvin*, and one AUV *Sentry* dive at the YBW-Sentry field (Fig. 6). Using these observations, we estimate $A_{d}$ using

Ad=B∫yminymax∫xminxmaxmaxi=1,2,…,N[(x−xi)2+(y−yi)2≤rd2]dxdy (3)

where *x* and *y* are hydrothermal chimney coordinates, $r_{d}$ is the mean radius of diffuse flow around each chimney, and *B* is a scaling factor used to account for the observation that diffuse flow occurs within small patches and not the entire area over which evidence for diffuse flow is observed (*17*, *22*). Taking the median of 168 individual measurements of the distance between indicators of diffuse flow and the nearest neighboring chimney, we find that $r_{d}\;=\;24\;\pm 12\;m$ . Within the entire diffuse flow field, only small portions of the area host the diffuse flow. To account for this partitioning, we estimate a scaling factor, *B*, by visually examining ~20,000 high-resolution (~23 MP) still images from seven submersible dives in the areas around active chimneys along the spreading segment. For each image frame, we estimated the proportion of the field of view occupied by indicators of diffuse flow, such as white sulfide precipitates or yellow bacterial mats [e.g., (*28*)], and in most analyzed images, diffuse flow evidence occupied 0.5 to 2% of the image. This percentage can increase to >5% in a few images around the YBW-Sentry field. Without comprehensive images of all diffuse vent fields and potential uncertainty from different view angles of still images, we cannot determine a single scaling factor with a confidence level. To avoid potentially overestimating the vent field area, we select the minimum scaling factor of 0.5%, i.e., *B* = 0.005, which is broadly consistent with previous results at the Lucky Strike hydrothermal field [Fig. 8; 0.5 to 1%; (*26*, *28*)].
