# Supplementary material for: Quantifying widespread hydrothermal chimneys on the East Pacific Rise flanks between 9°43′ and 57′N
Source: Sci Adv. 2025 Nov 7;11(45):eadv0788. doi: 10.1126/sciadv.adv0788 (PMC12594198; doi:10.1126/sciadv.adv0788)
Supplement: Supplementary file 1 — Supplementary Text Figs. S1 to S15 Tables S1 to S3 [file sciadv.adv0788_sm.pdf]

Science Advances
AAAS

Supplementary Materials for

Quantifying widespread hydrothermal chimneys on the East Pacific Rise flanks between 9°43′ and 57′N

Jyun-Nai Wu *et al.*

Corresponding author: Jyun-Nai Wu, jyun-nai.wu@whoi.edu

Sci. Adv. **11**, eadv0788 (2025)
DOI: 10.1126/sciadv.adv0788

This PDF file includes:

Supplementary Text
Figs. S1 to S15
Tables S1 to S3

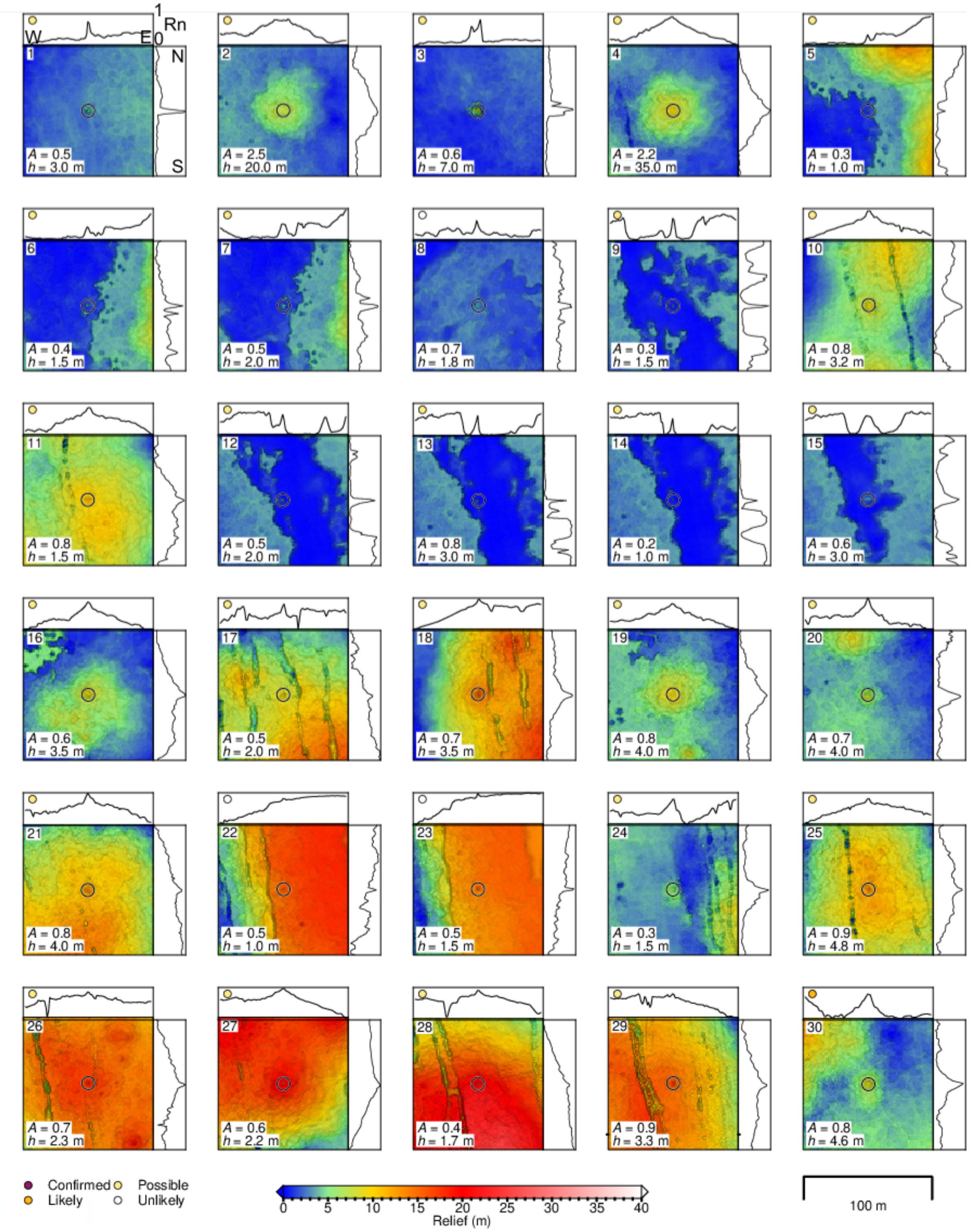

Figure S1 Maps of constructs 1-30. 100 x100 m map of construct, top panel and right panel show normalized bathymetric profile (Rn: normalized relief) across the construct from west to east and north to south, respectively. Dot and number at upper left corner of top panel show the category and order of constructs sorted from north to south, respectively; dot circle in the map is the construct; lower bottom corner of map noted values of A (aspect ratio) and h (height) of construct.

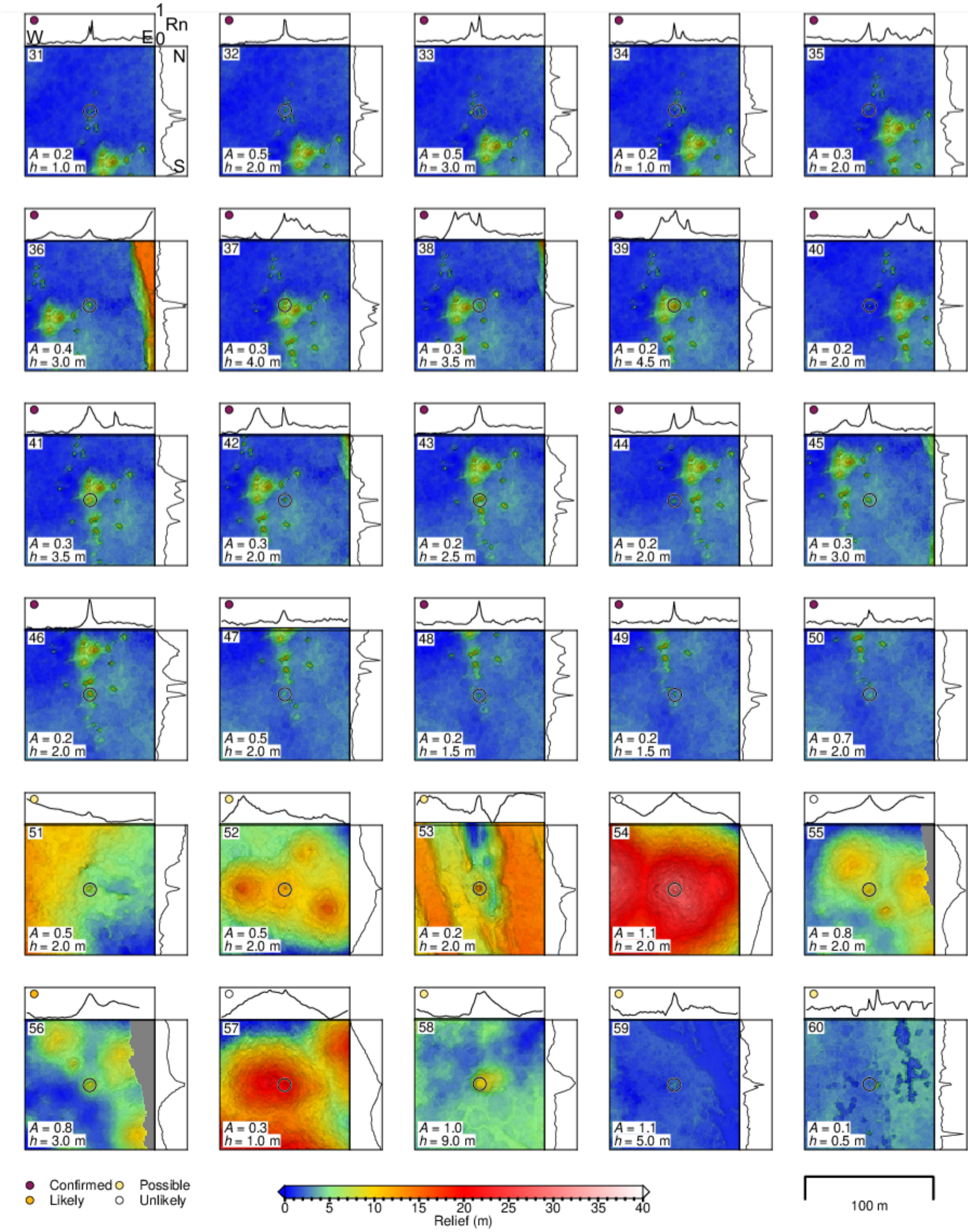

Figure S2 Maps of constructs 31-60. 100 x100 m map of construct, top panel and right panel show normalized bathymetric profile (Rn: normalized relief) across the construct from west to east and north to south, respectively. Dot and number at upper left corner of top panel show the category and order of constructs sorted from north to south, respectively; dot circle in the map is the construct; lower bottom corner of map noted values of A (aspect ratio) and h (height) of construct.

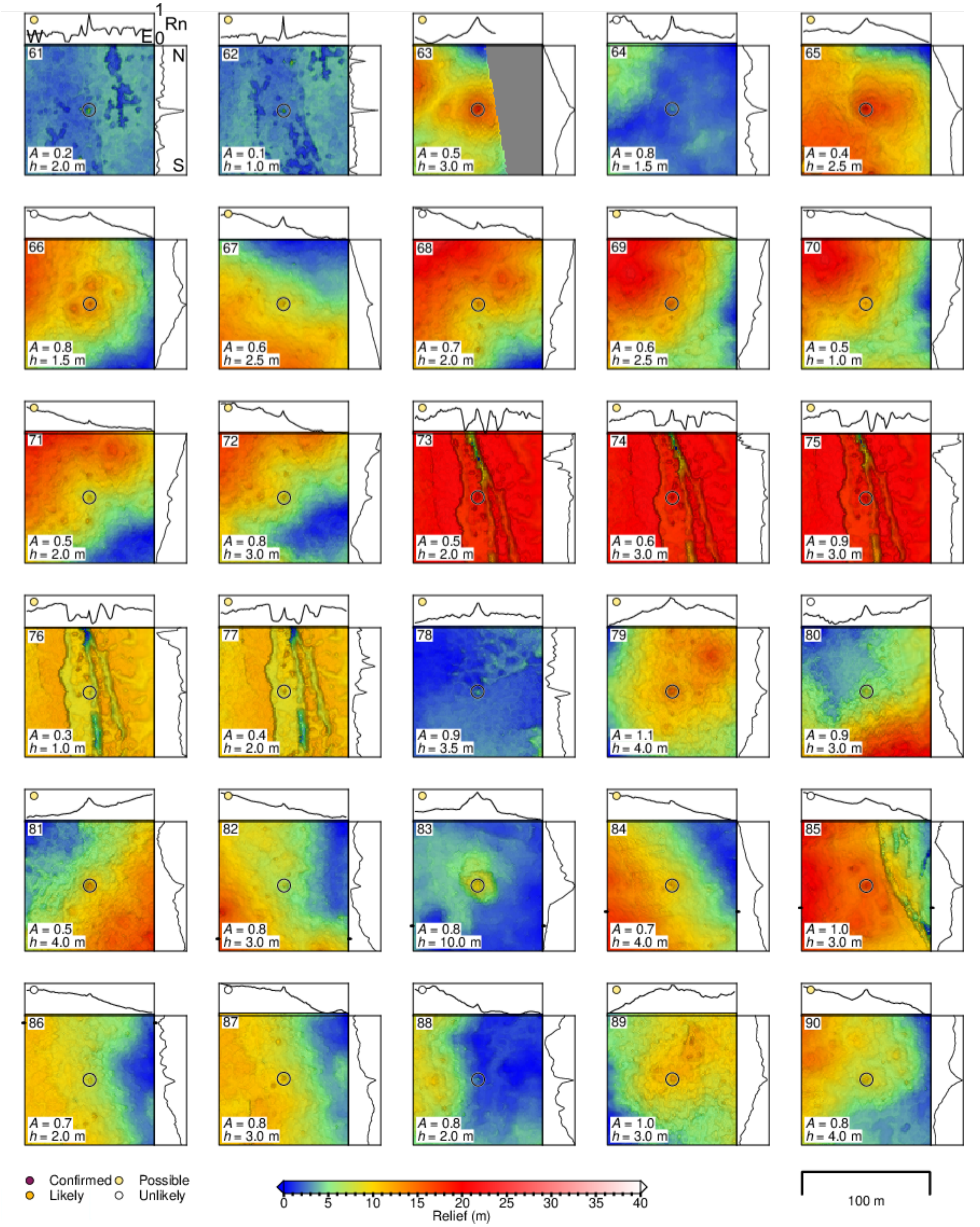

Figure S3 Maps of constructs 61-90. 100 x100 m map of construct, top panel and right panel show normalized bathymetric profile (Rn: normalized relief) across the construct from west to east and north to south, respectively. Dot and number at upper left corner of top panel show the category and order of constructs sorted from north to south, respectively; dot circle in the map is the construct; lower bottom corner of map noted values of A (aspect ratio) and h (height) of construct.

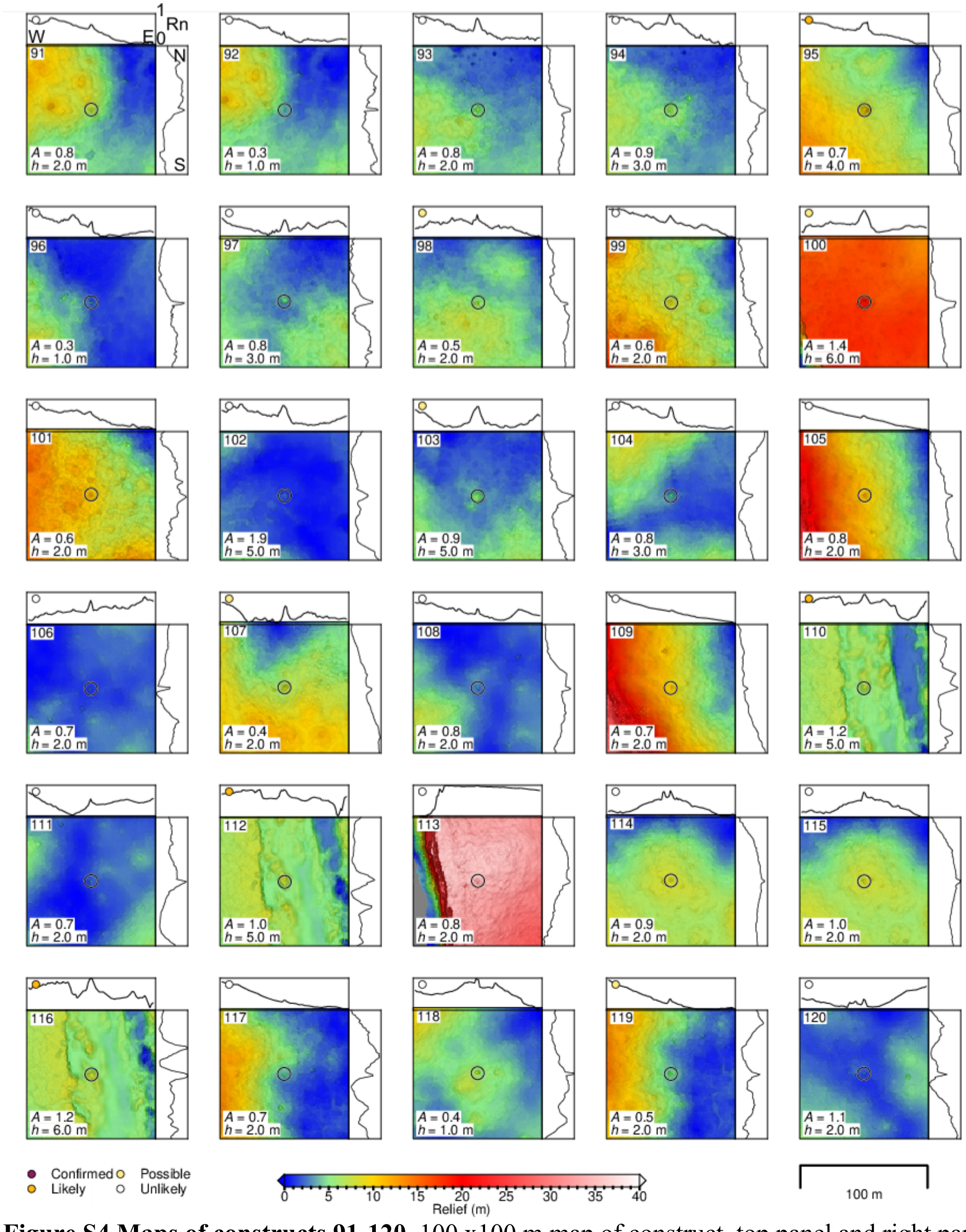

Figure S4 Maps of constructs 91-120. 100 x100 m map of construct, top panel and right panel show normalized bathymetric profile (Rn: normalized relief) across the construct from west to east and north to south, respectively. Dot and number at upper left corner of top panel show the category and order of constructs sorted from north to south, respectively; dot circle in the map is the construct; lower bottom corner of map noted values of A (aspect ratio) and h (height) of construct.

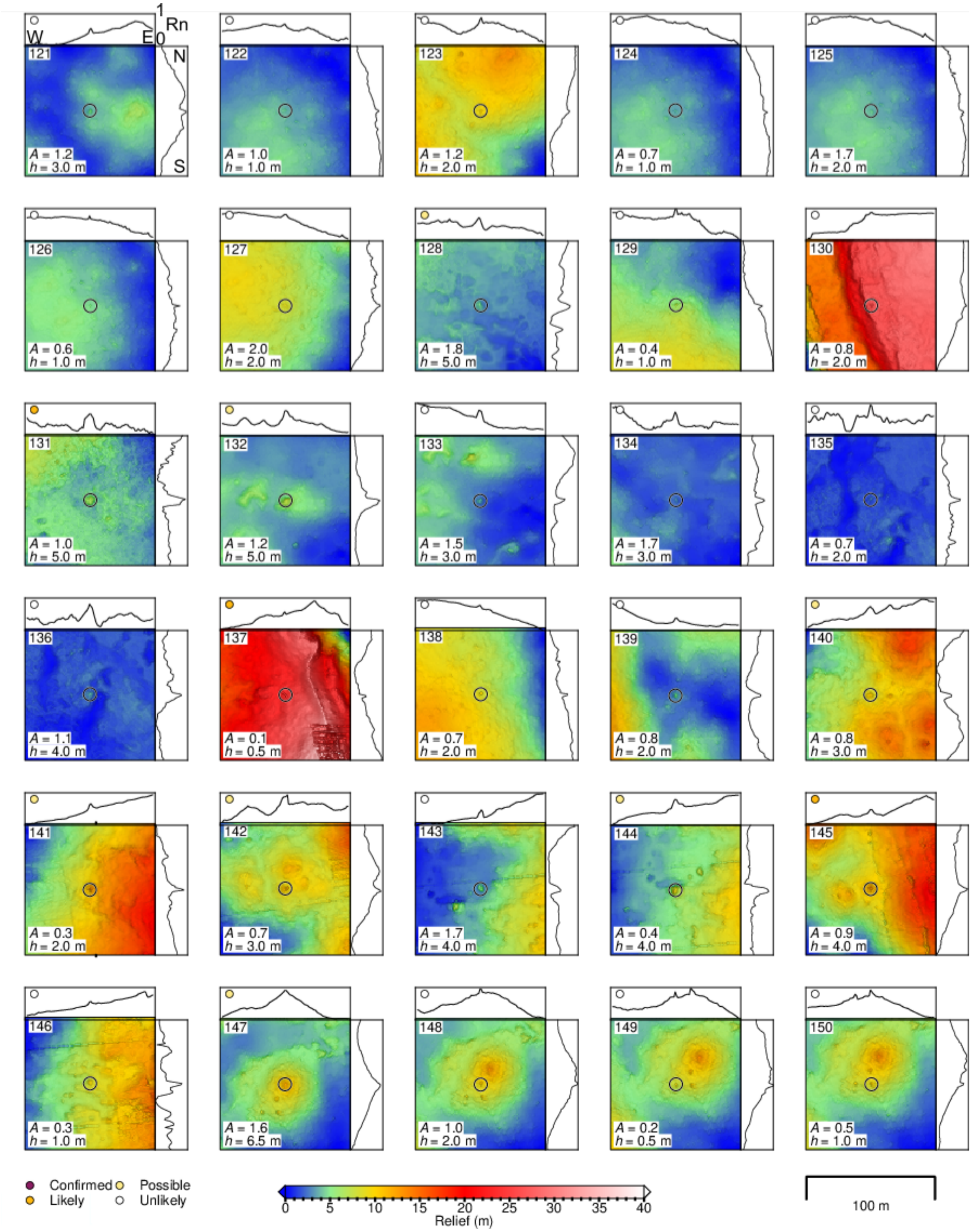

Figure S5 Maps of constructs 121-150. 100 x100 m map of construct, top panel and right panel show normalized bathymetric profile (Rn: normalized relief) across the construct from west to east and north to south, respectively. Dot and number at upper left corner of top panel show the category and order of constructs sorted from north to south, respectively; dot circle in the map is the construct; lower bottom corner of map noted values of A (aspect ratio) and h (height) of construct.

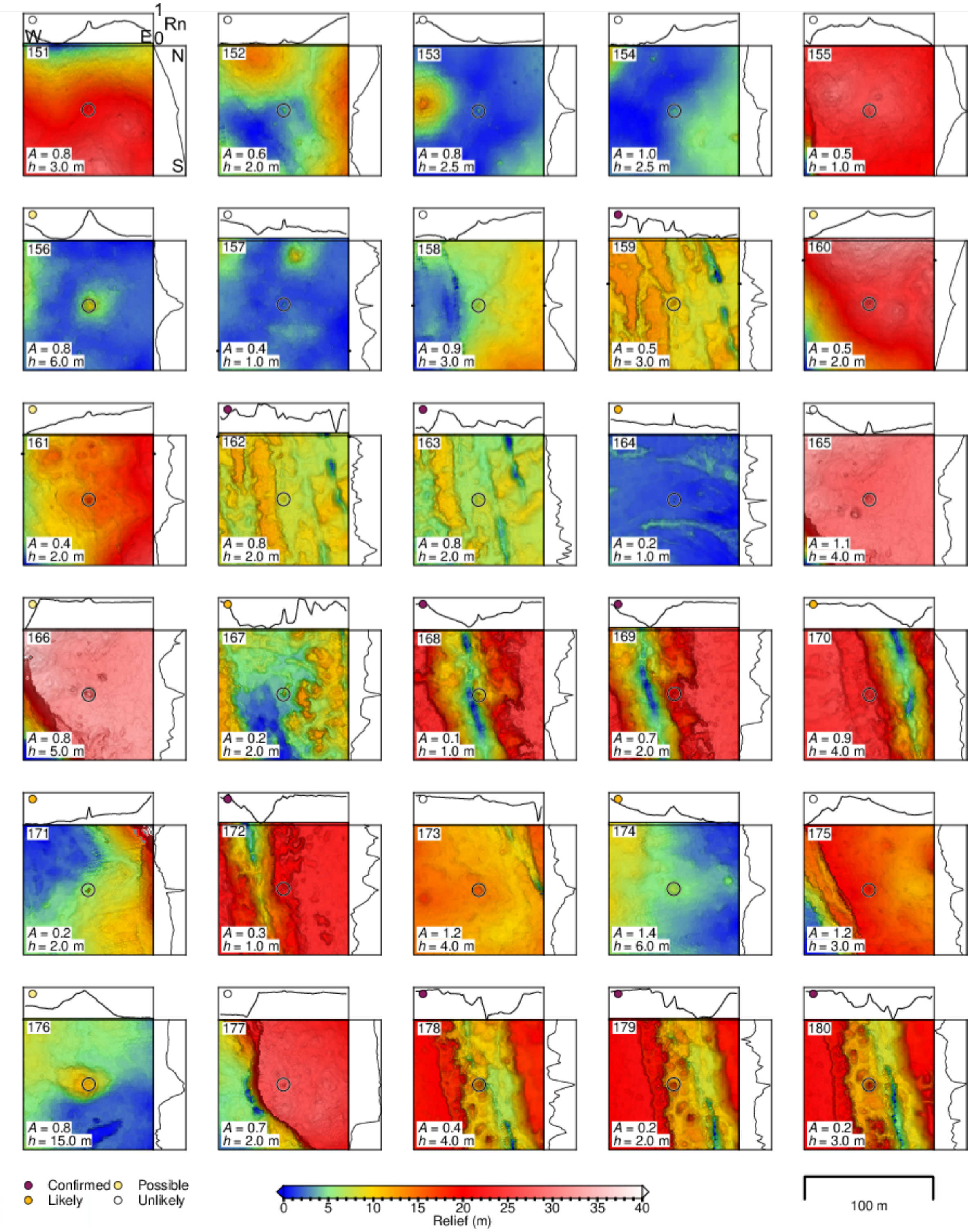

Figure S6 Maps of constructs 151-180. 100 x100 m map of construct, top panel and right panel show normalized bathymetric profile (Rn: normalized relief) across the construct from west to east and north to south, respectively. Dot and number at upper left corner of top panel show the category and order of constructs sorted from north to south, respectively; dot circle in the map is the construct; lower bottom corner of map noted values of A (aspect ratio) and h (height) of construct.

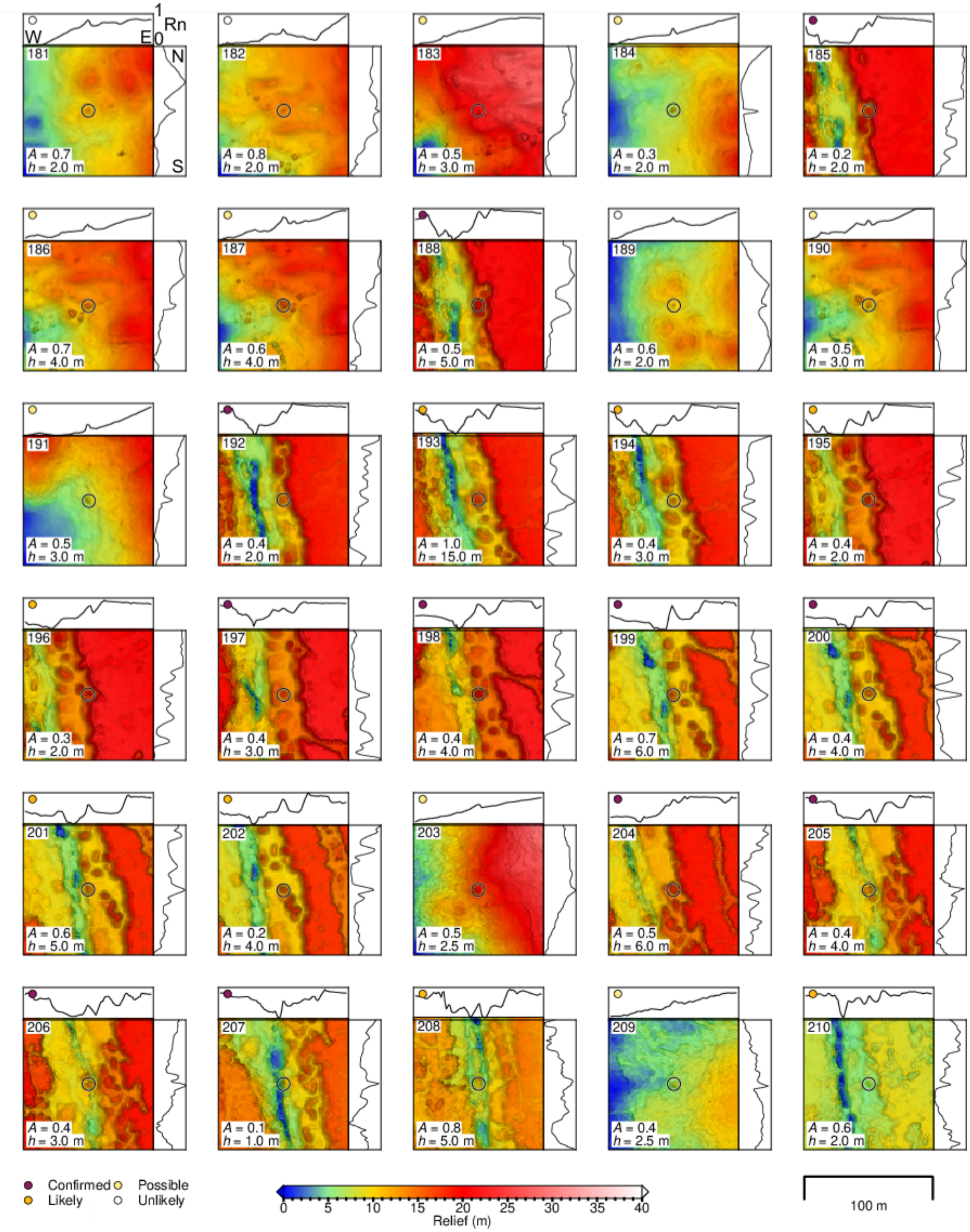

Figure S7 Maps of constructs 181-210. 100 x100 m map of construct, top panel and right panel show normalized bathymetric profile (Rn: normalized relief) across the construct from west to east and north to south, respectively. Dot and number at upper left corner of top panel show the category and order of constructs sorted from north to south, respectively; dot circle in the map is the construct; lower bottom corner of map noted values of A (aspect ratio) and h (height) of construct.

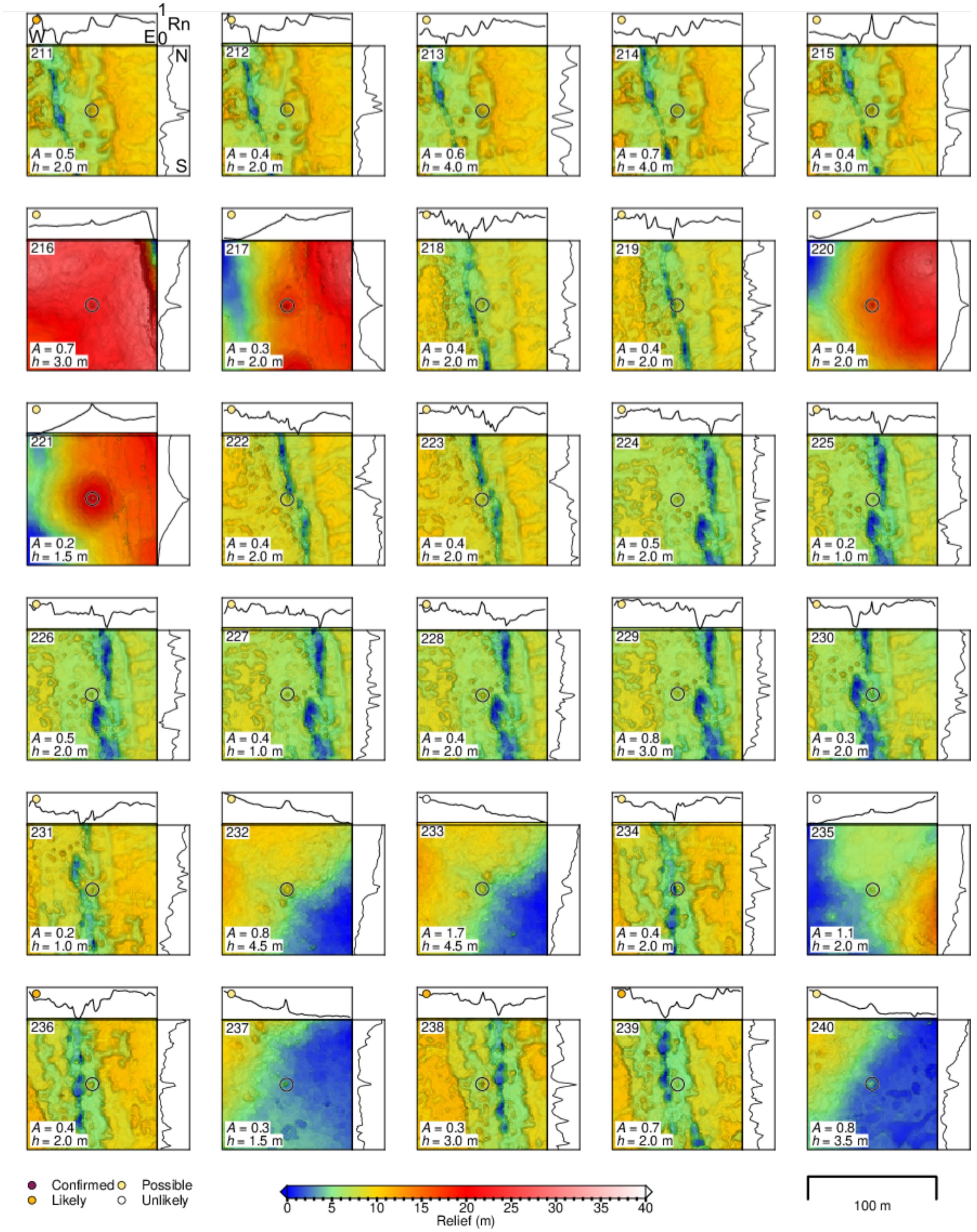

Figure S8 Maps of constructs 211-240. 100 x100 m map of construct, top panel and right panel show normalized bathymetric profile (Rn: normalized relief) across the construct from west to east and north to south, respectively. Dot and number at upper left corner of top panel show the category and order of constructs sorted from north to south, respectively; dot circle in the map is the construct; lower bottom corner of map noted values of A (aspect ratio) and h (height) of construct.

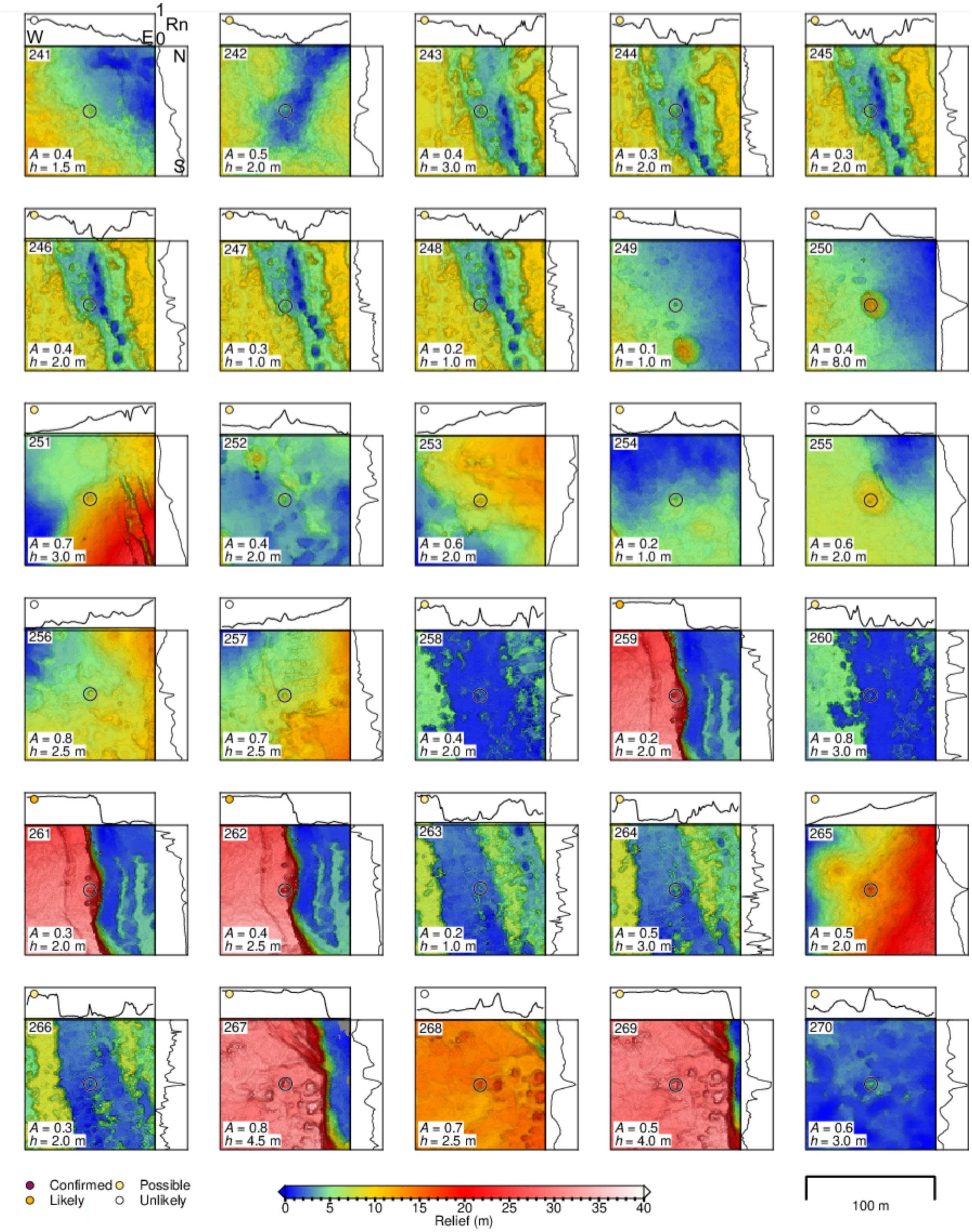

Figure S9 Maps of constructs 241-270. 100 x100 m map of construct, top panel and right panel show normalized bathymetric profile (Rn: normalized relief) across the construct from west to east and north to south, respectively. Dot and number at upper left corner of top panel show the category and order of constructs sorted from north to south, respectively; dot circle in the map is the construct; lower bottom corner of map noted values of A (aspect ratio) and h (height) of construct.

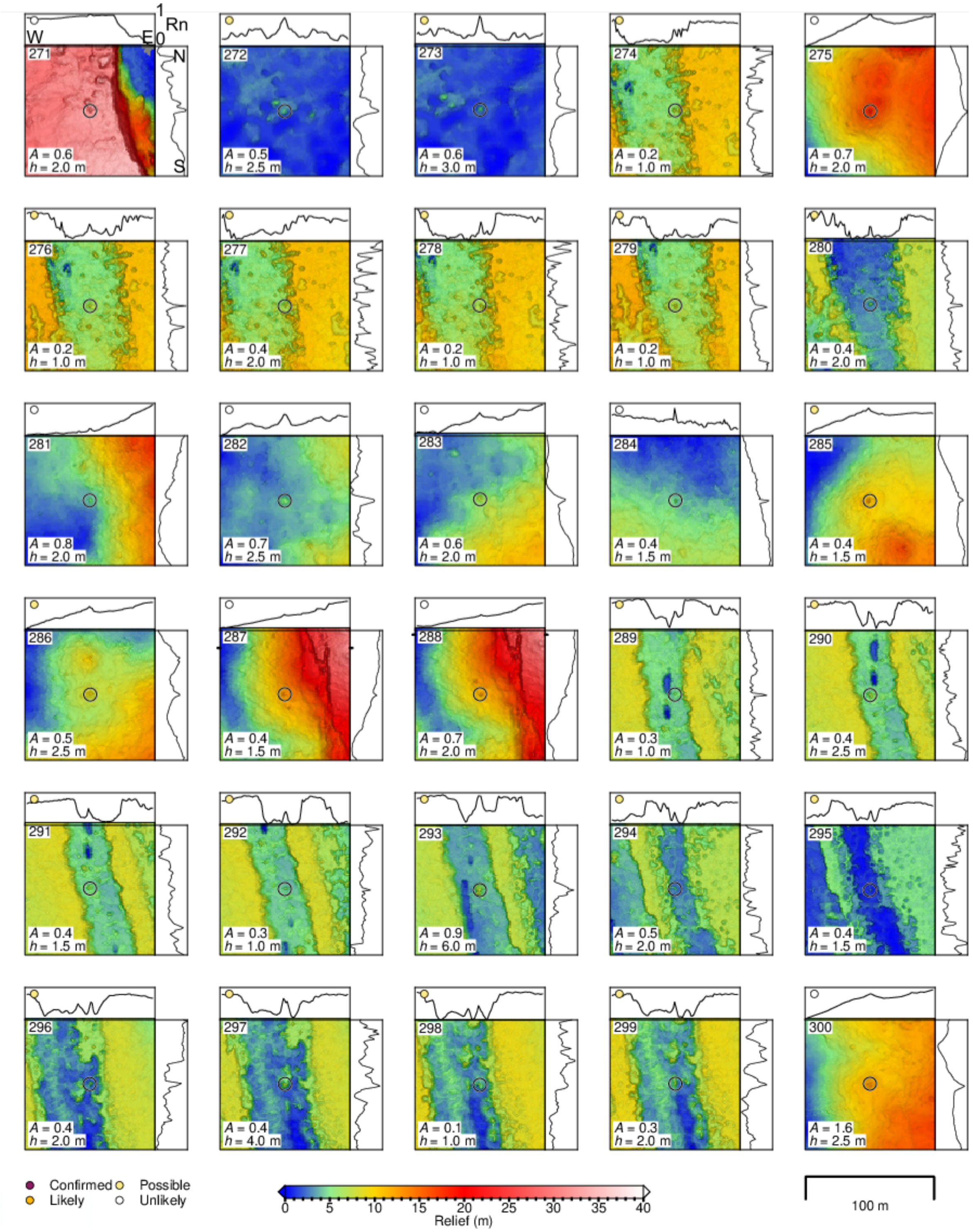

Figure S10 Maps of constructs 271-300. 100 x100 m map of construct, top panel and right panel show normalized bathymetric profile (Rn: normalized relief) across the construct from west to east and north to south, respectively. Dot and number at upper left corner of top panel show the category and order of constructs sorted from north to south, respectively; dot circle in the map is the construct; lower bottom corner of map noted values of A (aspect ratio) and h (height) of construct.

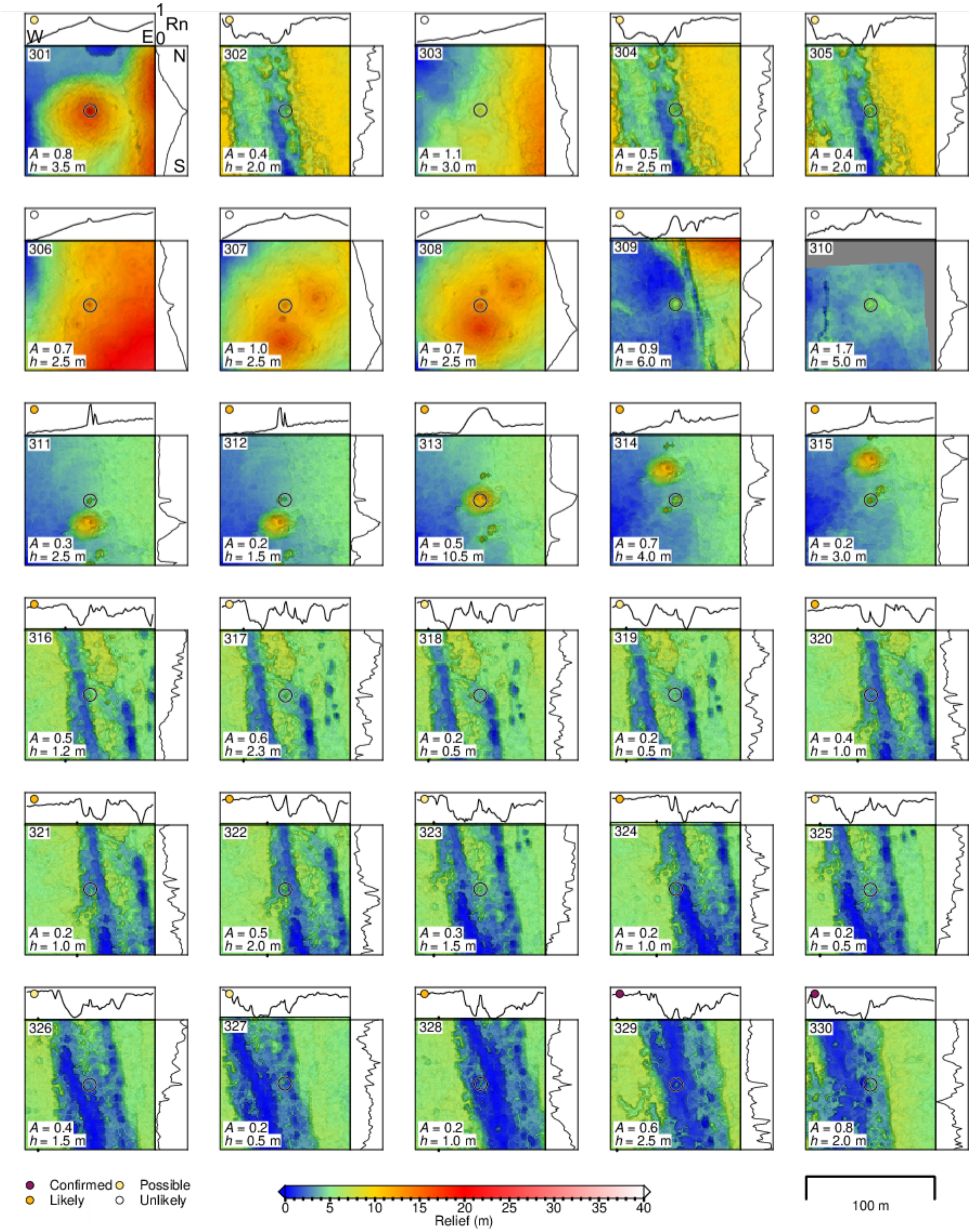

Figure S11 Maps of constructs 301-330. 100 x100 m map of construct, top panel and right panel show normalized bathymetric profile (Rn: normalized relief) across the construct from west to east and north to south, respectively. Dot and number at upper left corner of top panel show the category and order of constructs sorted from north to south, respectively; dot circle in the map is the construct; lower bottom corner of map noted values of A (aspect ratio) and h (height) of construct.

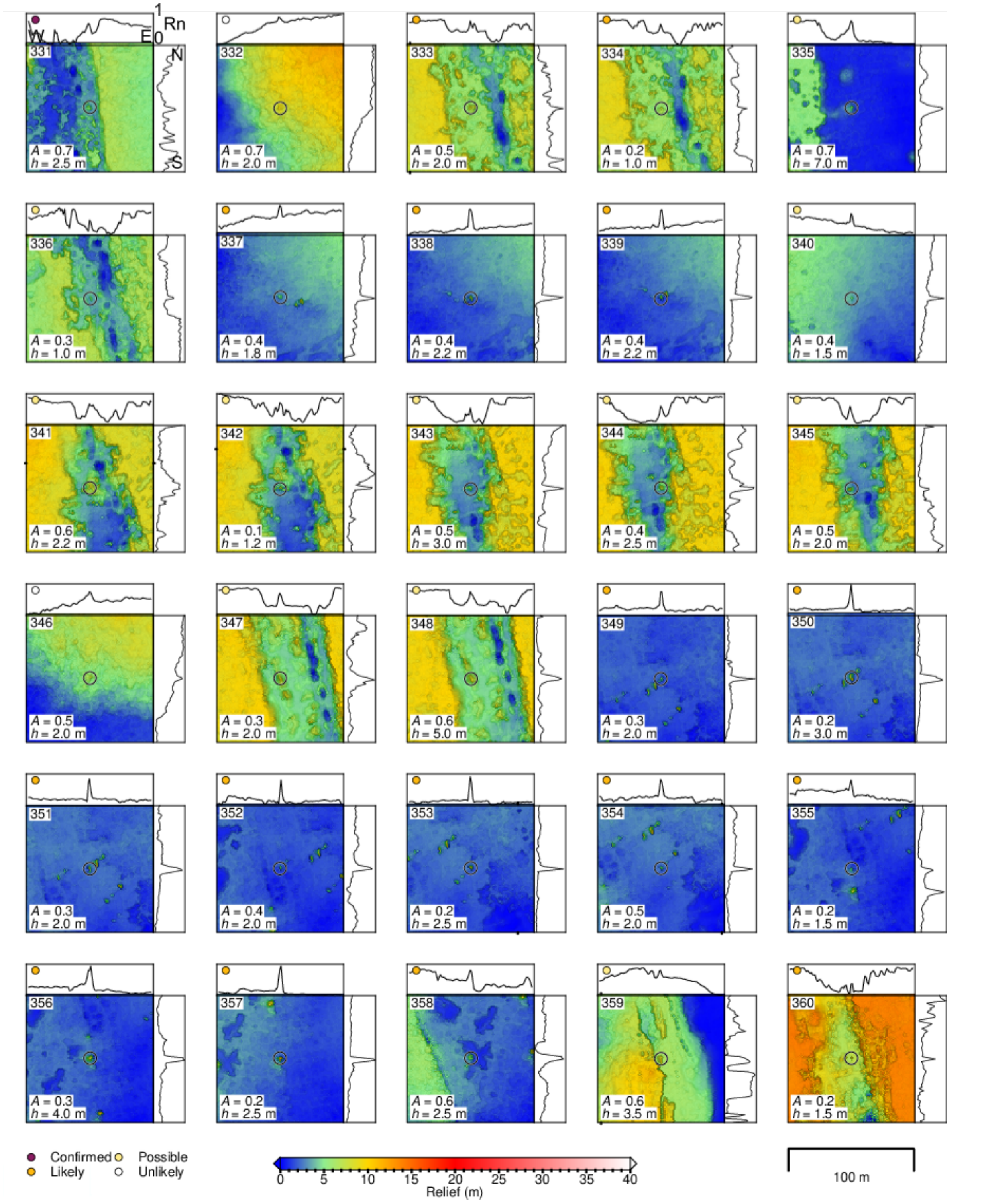

Figure S12 Maps of constructs 331-360. 100 x100 m map of construct, top panel and right panel show normalized bathymetric profile (Rn: normalized relief) across the construct from west to east and north to south, respectively. Dot and number at upper left corner of top panel show the category and order of constructs sorted from north to south, respectively; dot circle in the map is the construct; lower bottom corner of map noted values of A (aspect ratio) and h (height) of construct.

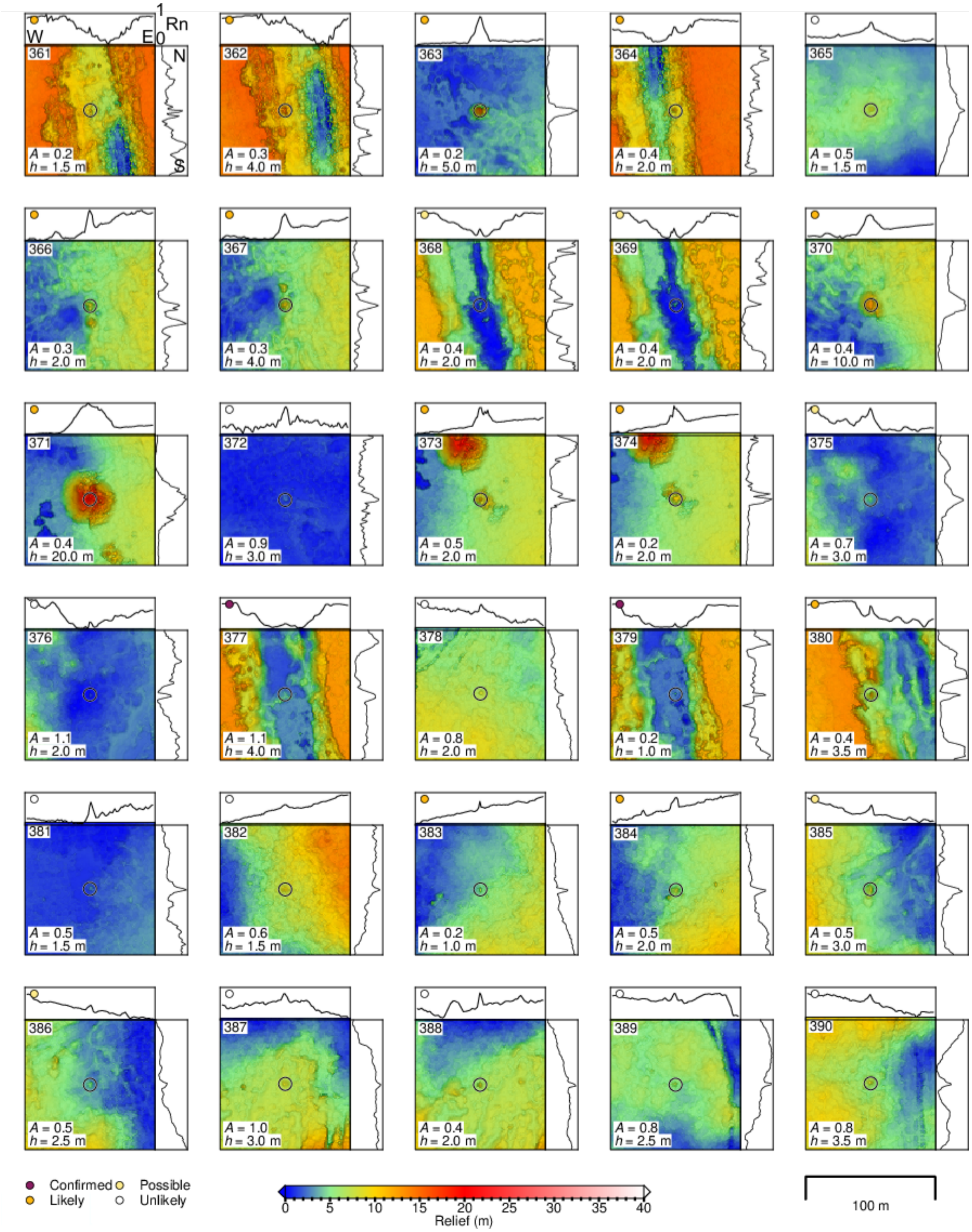

Figure S13 Maps of constructs 361-390. 100 x100 m map of construct, top panel and right panel show normalized bathymetric profile (Rn: normalized relief) across the construct from west to east and north to south, respectively. Dot and number at upper left corner of top panel show the category and order of constructs sorted from north to south, respectively; dot circle in the map is the construct; lower bottom corner of map noted values of A (aspect ratio) and h (height) of construct.

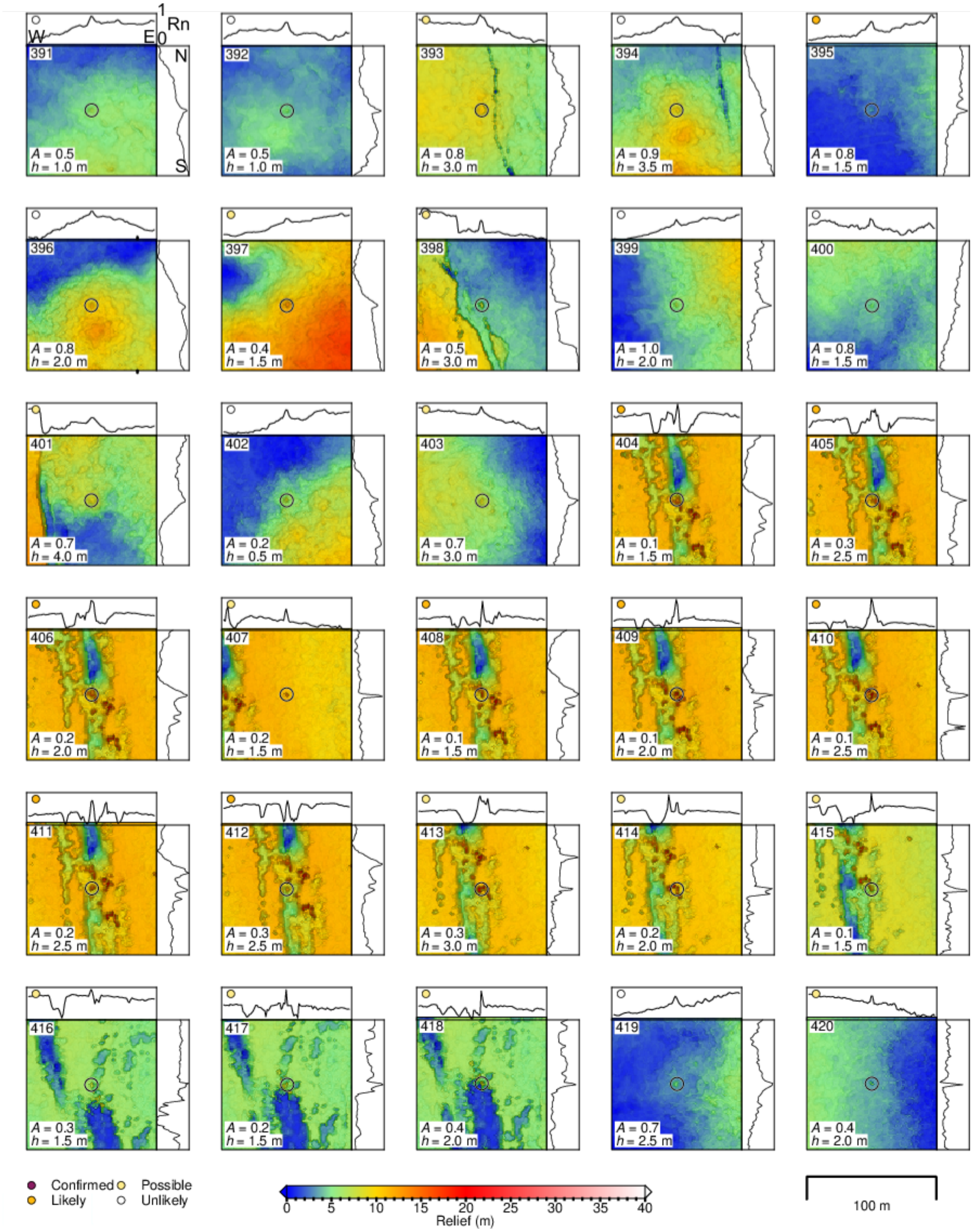

Figure S14 Maps of constructs 391-420. 100 x100 m map of construct, top panel and right panel show normalized bathymetric profile (Rn: normalized relief) across the construct from west to east and north to south, respectively. Dot and number at upper left corner of top panel show the category and order of constructs sorted from north to south, respectively; dot circle in the map is the construct; lower bottom corner of map noted values of A (aspect ratio) and h (height) of construct.

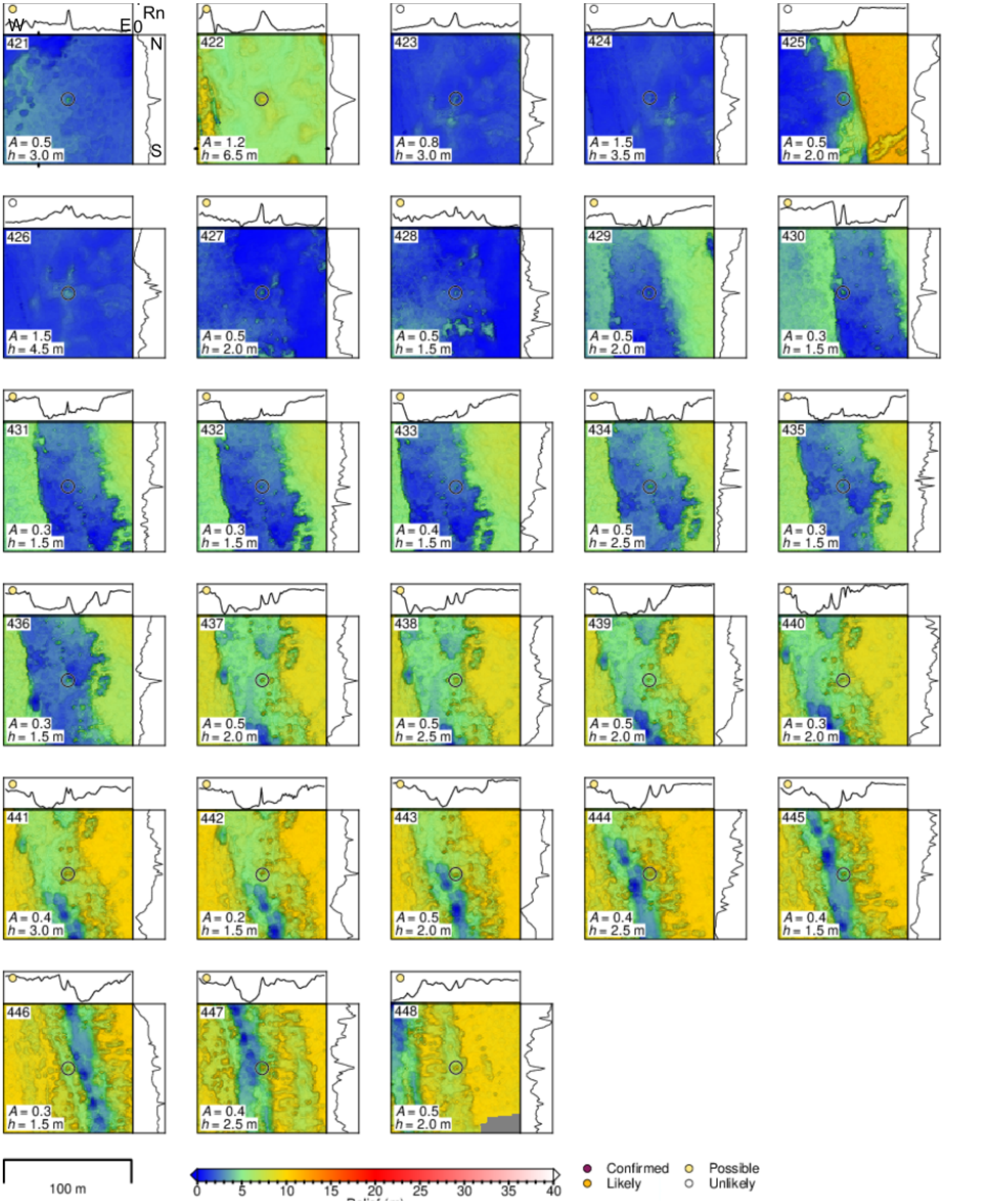

Figure S15 Maps of constructs 421-448. 100 x100 m map of construct, top panel and right panel show normalized bathymetric profile (Rn: normalized relief) across the construct from west to east and north to south, respectively. Dot and number at upper left corner of top panel show the category and order of constructs sorted from north to south, respectively; dot circle in the map is the construct; lower bottom corner of map noted values of A (aspect ratio) and h (height) of construct.

Table S1. Survey altitude of AUV *Sentry* dives

Sentry dive number	Survey altitude (m)
524	20
525	80
526	65
551	80
552	65
553	65
554	65
556	65
557	65
558	65
559	65
560	65
561	65
573	65
574	65
575	65
577	65
578	65
579	65
582	65

Table S2. Specification of sensors equipped on submersibles used in this study.

Instruments	Accuracy	Resolution
Oxygen sensor (Aanderaa Optode 4330F)	<2 μM	<0.1 μM
Pressure sensor (Paroscientific 8B7000-I)	0.01% of depth	10^{-8} m
Altimeter (Nortek DVL500)	0.1% of range	10^{-5} m

Table S3. Number of orifices at high-temperature vent sites

Site	Number of orifices	Median diameter (cm)
Bio9 (north)	2	5
Bio9 (Central)	2	5
Bio9 (South)	2	5
P vent	6	N/A
M vent	3	2
YBW (Northern)	11 in 5 structures	4
L vent	2	1
L hot 8	4	2
Tica	2	N/A
V vent	3	20
